# A Voluntary Statewide Newborn Screening Pilot for Spinal Muscular Atrophy: Results from Early Check

**DOI:** 10.3390/ijns7010020

**Published:** 2021-03-21

**Authors:** Katerina S. Kucera, Jennifer L. Taylor, Veronica R. Robles, Kristin Clinard, Brooke Migliore, Beth Lincoln Boyea, Katherine C. Okoniewski, Martin Duparc, Catherine W. Rehder, Scott M. Shone, Zheng Fan, Melissa Raspa, Holly L. Peay, Anne C. Wheeler, Cynthia M. Powell, Donald B. Bailey, Lisa M. Gehtland

**Affiliations:** 1 RTI International, Research Triangle Park, Durham, NC 27709, USA; vrobles@rti.org (V.R.R.); bmigliore@rti.org (B.M.); mlincolnboyea@rti.org (B.L.B.); kokoniewski@rti.org (K.C.O.); mduparc@rti.org (M.D.); mraspa@rti.org (M.R.); hpeay@rti.org (H.L.P.); acwheeler@rti.org (A.C.W.); dbailey@rti.org (D.B.B.J.); lgehtland@rti.org (L.M.G.); 2 American College of Medical Genetics and Genomics, Bethesda, MD 20814, USA; jtaylor@acmg.net; 3 Department of Pediatrics, The University of North Carolina at Chapel Hill, Chapel Hill, NC 27599-7487, USA; kclinard@email.unc.edu; 4 Department of Pathology, Duke University, Durham, NC 27710, USA; catherine.rehder@duke.edu; 5 North Carolina State Laboratory of Public Health, North Carolina Department of Health and Human Services, Raleigh, NC 27607, USA; scott.shone@dhhs.nc.gov; 6 Department of Neurology, University of North Carolina at Chapel Hill, Chapel Hill, NC 27599, USA; zheng_fan@med.unc.edu; 7 Department of Pediatrics and Department of Genetics, The University of North Carolina at Chapel Hill, Chapel Hill, NC 27599-7487, USA; powellcm@med.unc.edu

**Keywords:** newborn screening, spinal muscular atrophy, genetics, *SMN1* gene, pilot study

## Abstract

Prior to statewide newborn screening (NBS) for spinal muscular atrophy (SMA) in North Carolina, U.S.A., we offered voluntary screening through the Early Check (EC) research study. Here, we describe the EC experience from October 2018 through December 2020. We enrolled a total of 12,065 newborns and identified one newborn with 0 copies of *SMN1* and two copies of *SMN2*, consistent with severe early onset of SMA. We also detected one false positive result, likely stemming from an unrelated blood disorder associated with a low white blood cell count. We evaluated the timing of NBS for babies enrolled prenatally (*n* = 932) and postnatally (*n* = 11,133) and reasons for delays in screening and reporting. Although prenatal enrollment led to faster return of results (median = 13 days after birth), results for babies enrolled postnatally were still available within a timeframe (median = 21 days after birth) that allowed the opportunity to receive essential treatment early in life. We evaluated an SMA q-PCR screening method at two separate time points, confirming the robustness of the assay. The pilot project provided important information about SMA screening in anticipation of forthcoming statewide expansion as part of regular NBS.

## 1. Introduction

Early Check (EC) is a comprehensive opt-in newborn screening (NBS) framework that supports pilot studies of conditions not yet implemented in state NBS. The overarching objective is to demonstrate the feasibility and acceptability of statewide screening and follow-up for inborn conditions, inform public policy, and support U.S. Recommended Uniform Screening Panel (RUSP) nominations [1]. The project is a collaboration between RTI International, the North Carolina State Laboratory of Public Health (NCSLPH), and three major universities—Duke University, University of North Carolina at Chapel Hill, and Wake Forest University. EC launched screening for spinal muscular atrophy (SMA) in October 2018 and will continue until the spring of 2021. The EC experience with screening for SMA over the past 26 months, together with screening for fragile X syndrome (FXS) and more recently for Duchenne muscular dystrophy (DMD), illustrates the utility of the framework as a robust platform amenable to a variety of NBS pilot studies.

SMA is caused by a deficiency of the survival of motor neuron (SMN) protein, resulting in progressive degeneration and irreversible loss of the anterior horn cells in the spinal cord and the brain stem nuclei. Without treatment, the decreased level of the SMN protein leads to muscle weakness, debilitating atrophy, and life-limiting lung disease [2].

A majority of the required SMN protein is produced by the *SMN1* gene, which is a part of a 500 kb inverted duplication on 5q13, containing repetitive elements prone to rearrangements and deletions. Homozygous loss of *SMN1* at exon 7 is common to >95% of SMA cases [3] and can be detected by the highly sensitive molecular assay now frequently utilized in NBS. Mutations in the centromeric paralog *SMN2* are not considered pathogenic [4]; however, *SMN2* is an important disease modifier [5]. The severity of the disease is frequently inversely correlated with *SMN2* copy number [5,6]; thus, detection of *SMN2* copy number is performed by some NBS programs and at confirmatory testing to inform treatment decisions.

Variations of a quantitative polymerase chain reaction (qPCR)-based assay that target exon 7 of *SMN1* have been used by other pilot studies [7,8,9,10,11,12] and state NBS programs [13,14]. More recently, multiplex assays have been developed to allow for simultaneous screening for SMA and severe combined immunodeficiency (SCID) [15], thereby lowering the cost, eliminating the need for an additional dried blood spot (DBS) punch and reducing the cumulative time to reporting when implemented on a statewide scale.

Screening for SMA has been piloted and/or implemented in Taiwan, Australia, Germany, Belgium, China, Canada, Japan, and the United States resulting in early identification of infants with SMA and leading to presymptomatic treatment and an opportunity to investigate the effects of new and emerging therapies [16]. In the United States, SMA was added to the RUSP in July 2018 [17] and as of January 2021, over half of the states have implemented NBS for SMA [18]. Here, we report on optional NBS for SMA, which was implemented prior to state-mandated universal screening and thus required parental consent. We describe a generalizable timeline from birth to screening, clinical evaluation, and treatment for enrolled newborns.

## 2. Materials and Methods

### 2.1. Study Recruitment

The SMA pilot was a part of the EC framework, which is in its entirety designed as a voluntary study. EC is overseen by the University of North Carolina at Chapel Hill Institutional Review Board (#18-0009). Parents enrolled their infants via an electronic research portal. Enrollment was offered prenatally, at or after 13 weeks gestation, and postnatally until the fourth week postpartum [1]. Several strategies were employed to inform parents of eligible newborns about the study: (1) the North Carolina Department of Health and Human Services sent a letter and a study flyer to the mothers of all newborns who received NBS in North Carolina and for whom there was an address on the NBS DBS card; (2) invitations with a link to the EC electronic consent portal were sent through My Chart electronic health record patient portals at Duke University and University of North Carolina at Chapel Hill to eligible pregnant women; (3) social media organic posts and paid ads targeted to pregnant women living in North Carolina were posted on Facebook and Instagram in both English and Spanish; and (4) informational materials were distributed through select hospitals, clinics, and the North Carolina Special Supplemental Nutrition Program for Women, Infants, and Children.

### 2.2. Assay Validation

Two assay validation studies were performed. The first was completed at the NCSLPH prior to the start of the pilot study in October 2018 and the second was completed before relaunch at the time of EC laboratory move to RTI in April 2020.

### 2.3. Specimens

Normal control from human newborn cord blood and two SMA-positive and two carrier controls made from leukocyte-depleted blood enriched with SMA and SMA carrier cell lines were provided by the U.S. Centers for Disease Control and Prevention (CDC). *SMN1* copy numbers were confirmed with droplet digital PCR (ddPCR). No template control (NTC) was a blank piece of filter paper. Thirty patient and carrier specimens from older individuals aged 4–83 with known *SMN1* copy numbers were obtained from Biogen to assess accuracy. A North Carolina-specific reference range was established twice, using 1982 retrospective routine de-identified DBS specimens from NCSLPH in the first validation and 736 previously tested EC NBS specimens in the second validation. For SMA screening, residual blood spots from NC routine screening were matched to consents after all state NBS was complete, the DBS card data were imported into the EC LIMS system, and the retrieved specimens were punched and tested daily at the NCSLPH by RTI staff for the first 18 months and at RTI for the remainder of the study.

### 2.4. SMA Screening Assay

A modified version of the real-time TaqMan qPCR assay [15] was used to detect the presence or absence of the homozygous deletion of *SMN1* at exon 7. For each specimen, one 1.5 mm DBS punch was placed in a 96-well plate and washed twice with 90 µL of Extracta DNA Prep (QuantaBio. Cat. No. C95171-500, Beverly, MA, USA) with shaking at 2500 rpms, initially for 10 min, then for 5 min, discarding the eluate after each shake. Washed DBS punches were left in the wells and 15 µL PCR reaction mix was added (PerfeCTa^®^ Multiplex qPCR ToughMix, Cat. No. 95140-05K, QuantaBio, Beverly, MA, USA). The *SMN* primers amplify both *SMN1* and *SMN2* at exon 7. The probe targets *SMN1*. *RPP30* served as an internal control. *SMN* Forward Primer, 5′-CTT GTG AAA CAA AAT GCT TTT TAA CAT CCA T-3′ (900 nM); *SMN* Reverse Primer, 5′-GAA TGT GAG CAC CTT CCT TCT TTT T-3′ (900 nM); *SMN1* Probe, 5′-/5CY5/GGT T+T+C +A+G+A+CAA/3IAbRQSp/-3′ (60 nM); *RPP30* Forward Primer, 5′-AGA TTT GGA CCT GCG AGC G–3′ (60 nM); *RPP30* Reverse Primer, 5′-GAG CGG CTG TCT CCA CAA GT -3′ (150 nM); *RPP30* Probe, 5′-/5HEX/TTC TGA CCT/ZEN/GAA GGC TCT GCG CG/3IABkFQ/-3′ (200 nM) (Integrated DNA Technologies, Glendale, CA, USA). The assay was run on two AriaMx G8830A Real-Time PCR Systems (Agilent, Santa Clara CA, USA) for the first 18 months of the study (conducted at the NCSLPH) and on two QuantStudio 7 Pro Real-Time PCR Systems (ABI, Foster City, CA, USA) for the remainder of the study (at RTI). Thermal cycling: 1 cycle at 45 °C or 5 min, 1 cycle at 95 °C for 20 min, and 45 cycles of (95 °C for 15 s and 60 °C for 1 min). A fixed amplification signal was used to assess Cq.

### 2.5. Screening, Reporting, Confirmatory Testing, and Follow-Up

**Figure 1:**
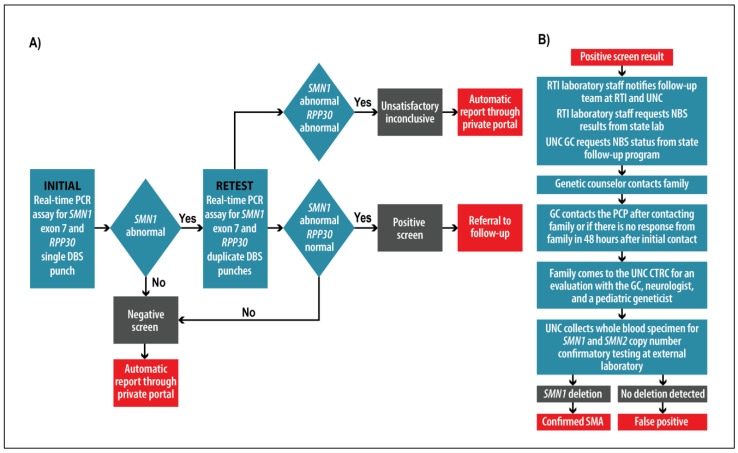
(**A**) Screening and (**B**) follow-up algorithms.

SMA testing was prioritized over other EC tests (FXS and DMD) to ensure that sufficient specimen was available and to expedite critical reporting. The screening algorithm (Figure 1A) involved initial testing in singlicate and retesting in duplicate for specimens above the initial cut-off. A typical batch was 15–60 newborn specimens run together with no template control (NTC), and controls with 0, 1 and 2 copies of *SMN1* and normal *RPP30*. Screen-negative SMA results were held and reported together with other EC study results (FXS and DMD). Parents of all screen-negative infants were notified via email and directed to an online portal to view the results. Screen-positive results were reported by the laboratory staff to the clinical genetics follow-up team immediately after the retest (Figure 1B). Prior to reporting to parents, the laboratory staff inquired with the NCSLPH regarding results from routine state NBS and regarding repeat specimens, and the genetic counselor inquired with the state’s follow-up program to ensure reciprocal knowledge and coordinated reporting in the case of a “double positive” for EC and state NBS.

Parents of screen-positive infants were contacted by a genetic counselor and referred for evaluation by a medical geneticist and a pediatric neurologist. Confirmatory specimens were collected at the initial clinical evaluation appointment and sent to an external laboratory for confirmatory testing. Parents were provided with educational materials, access to support organizations, and counseling about treatment options.

Baseline assessment of infant needs, developmental functioning, and parental mental health was conducted as a part of EC short-term follow-up and additional consent was obtained for engagement past 6 months of age. Screen-positive infants were assessed for potential delays in early cognitive, language, and motor functioning, along with early temperament/behavior, feeding, and sleep challenges. Parental mental health and family well-being was assessed through measures of postpartum depression, anxiety, general health, perceived social support, and parenting stress. An EC long-term follow-up program provides continued monitoring, including provision of information, support, developmental and medical surveillance, and treatment referrals for all screen-positive infants and their families [1].

## 3. Results

### 3.1. Validation Study

Validation experiments consistently showed *SMN1* amplification for SMA carriers and normal controls (negative result) and no amplification for SMA patients (positive result). The two validation studies yielded comparable precision results for those patient and carrier specimens which were used in both studies (Table S1), confirming that the control materials and assay performance had been stable for the 18 months. No carryover was observed in either validation study.

An initial cut-off for *SMN1* was set at Cq ≥ 26 corresponding to 99.7% of the Cq values from the first and 99.9% from the second population study below the cut-off. All newborn samples testing above the initial cut-off were retested in duplicate. Those at or above the final cut-off (Cq ≥ 27) with *RPP30* Cq < 26 were considered screen-positive and were referred for follow-up. Specimens below the final cut-off *SMN1* Cq < 27 were reported as screen-negative. Specimens with *SMN1* Cq ≥ 27 and *RPP30* Cq ≥ 26 were reported as unsatisfactory (Table 1).

There was a slight shift of the *SMN1* Cq distribution between the initial and second validation study toward higher Cq values (Figure S1, Table S2); however, both distributions were within the distribution of *SMN1* Cq values from the entire EC screened population (Figure S1C) and the shift did not affect the cut-off.

### 3.2. Screening for SMA

**Figure 2:**
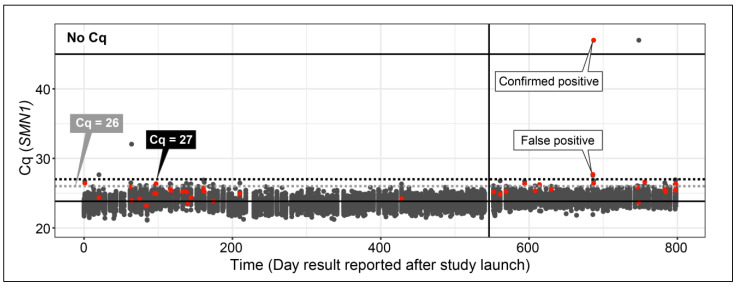
qPCR Cq values for newborn specimens screened for SMA by the EC study between October 2018 and December 2020. Initial testing (gray dots) was performed with a single 1.5 mm DBS punch. Specimens above initial cut-off Cq ≥ 26 (gray dotted line) were retested in duplicate (red dots). Specimens with repeat mean *SMN1* Cq ≥ 27 and *RPP30* Cq < 26 were reported as screen-positive at follow-up. Gray dots above the cut-off indicate initial results that resolved on retest. Reporting of negative results was paused for 7 days during the lab move, shown by a gap in the data and a vertical line at ~550 days after study launch. Solid horizontal black lines indicate overall mean from all screens Cq = 23.84 and maximum number of qPCR cycles used Cq = 45. Results shown by dots above Cq = 45 had a “No Cq” value.

The 12,065 newborns enrolled in EC (Table 2 and Figure 2) represented ~5% of the NC newborn population and all but one North Carolina county as well as some border counties in South Carolina, particularly those surrounding the Charlotte, NC, area.

Two specimens were not tested because of insufficient quantity. A total of 36 specimens were above the initial *SMN1* cut-off (Cq ≥ 26). Two of those retested above the final *SMN1* cut-off (Cq ≥ 27) and were reported as screen-positive. In total, 12,061 normal results, 2 screen-positive results, and 2 unsatisfactory results were reported.

### 3.3. Factors Affecting Timing of Early Check Screening

**Figure 3:**
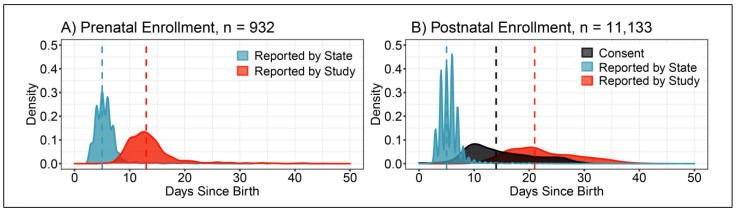
Time to the return of EC results, density plots. Values are given in days post birth. Birth to return of results for (**A**) prenatal (*n* = 932) and (**B**) postnatal (*n* = 11,133) consents. Birth date enrollments are included in postnatal consents. Vertical dashed lines represent median time post birth for each density plot. NBS results returned by the state (blue, the median for both prenatal and postnatal consents was 5 days). Results returned by EC (red, the median for prenatal and postnatal consents was 13 days and 21 days respectively). Consents obtained by EC (black, the median for prenatal consents was 87 days before birth (not shown) and postnatal consents 14 days after birth).

The median time of return of results from routine state NBS was 5 days after birth (Figure 3). The median return of the study results for babies enrolled prenatally was 13 days after birth, and 21 days after birth for babies enrolled postnatally.

The median time of prenatal consent was 87 days before birth (*n* = 932) and of postnatal consent 14 days after birth (*n* = 11,133). Factors affecting timing independent of the EC protocol are described in Table S3. The mean time interval between matching specimens (after becoming available from the state) to EC consents and reporting results to parents was 5.47 ± 2.32 days (*n* = 932) and 4.68 ± 2.64 days (*n* = 11,133) for prenatal and postnatal enrollment respectively. The turnaround time is inflated by the fact that results for SMA screen-negative babies were held and reported together with results for FXS and DMD, for which screening occurs after screening for SMA and is not performed daily.

### 3.4. Case Studies

#### 3.4.1. Case #1, Borderline/False Positive SMA Screen

The mother consented to EC screening 17 days after birth (Table 3). A positive NBS result for SMA was reported by the EC 26 days after birth (*SMN1* Cq = 27.70 and *RPP30* Cq = 25.06). Other EC tests and the state NBS panel were negative except for an initial borderline SCID result. Repeat DBS specimen tested negative for SCID by the state. Repeat specimens testing is not a part of the EC screening algorithm (Figure 1); however, the study team concluded that the result could provide additional information to the family. The repeat SMA result was unsatisfactory because of the *RPP30* value above the cut-off; however, the result was similar to the first specimen (Figure 2), suggesting a likely false positive. Both specimens were later tested by the NCSLPH with an SMA/SCID multiplex assay using DNA extracted from 3.2 mm punches with similar *SMN1* results [19].

A clinical evaluation at 30 days after birth indicated normal physical and neurological exams. Multiple blood tests indicated potential autoimmune neutropenia of infancy, and the infant was referred to pediatric hematology for further evaluation. Carrier testing of parents’ saliva indicated that one parent is a carrier for SMA (single copy of *SMN1* at exon 7) and the other is negative. Residual risk for SMA remained as the “negative” parent may be a carrier for another mutation not detected by the *SMN1* exon 7 deletion assay. A ddPCR test by an external laboratory on the proband’s initial venous blood sample detected a single copy of *SMN1* consistent with carrier status. The baby was later diagnosed with Shwachman-Diamond Syndrome, a rare genetic disorder that affects bone marrow and the generation of white blood cells. Follow-up for SMA has been discontinued. The family continues to stay in contact with the EC follow-up team and has been offered participation in long-term follow-up activities.

#### 3.4.2. Case #2, Positive SMA Screen, Confirmed 0 Copies of *SMN1* and 2 Copies of *SMN2*

The mother consented to EC screening 1 day prior to birth (Table 3). A positive NBS result for SMA (*SMN1* Cq = “No Cq” and *RPP30* Cq = 23.10) was reported by EC 10 days after birth. Other EC tests and the state NBS panel were negative. Both parents were known carriers. The mother had non-invasive prenatal testing for SMA that indicated decreased risk (from 1:4 to 1:40) for the fetus to be affected with SMA. The parents chose to enroll in EC in part for confirmation.

Physical and neurological exams performed on the newborn 13 days after birth noted a mild head lag. An external laboratory confirmed a homozygous deletion of *SMN1* with two copies of *SMN2* (MLPA-seq), consistent with a severe infantile onset form of SMA. Second clinical evaluation at 21 days post birth noted trace patellar deep tendon reflex, mild head lag, mild hypotonia, and mild tachypnea suggestive of early manifestation.

The child was feeding well and gaining weight. The parents had been interested in onasemnogene abeparvovec-xioi (Zolgensma); however, the infant did not qualify due to AAV9 antibody titer result >1:200 (<1:50 is required for onasemnogene abeparvovec-xioi [20]). The infant was started on the first of four loading doses of nusinersen (Spinraza) (12 mg each) on day 30 of life. Subsequent doses were administered at 6, 8, and 12 weeks. The patient showed increasing motor weakness and reduced movement of the limbs (lower extremities worse than upper extremities), worsening head lag, hypotonia, areflexia, and tachypnea at age 30 days and at 6 weeks. At 8 weeks the patient showed clinical improvement and scored 45/64 on CHOP INTEND and 4/26 on HIINE and continued to improve at 12 weeks of age. Repeated AAV9 antibody titer remained >1:200. The prevalence of neutralizing antibodies to AAV is typically moderate at birth and the lowest at the age of 7–11 months [21]. Another AAV9 test is planned at the next nusinersen dosing at the age of 7 months. This is the first confirmed case of a baby with SMA identified by a NBS pilot study in North Carolina.

## 4. Discussion

We employed several pre- and postnatal recruitment strategies, all without an in-person contact, to reach broadly across the population and maximize equity in participation. Although prenatal enrollment offers the greatest potential benefit to timely diagnosis and treatment, it is more challenging, because EC has no access to contact information of expectant mothers statewide. Postnatal recruitment had the advantage of reaching all birthing mothers in the state for whom contact information was available; however, the timing presents some challenges, such as capturing the interest of new mothers who are focused on newborn care and delays in testing because of later enrollment.

The statewide recruitment and prenatal consent are unique to EC. These strategies proved to be effective even throughout the COVID-19 pandemic, during which in-person access to parents has been severely limited. However, the enrollment volume (~5% of the state’s births) demonstrates the major challenge of implementing a feasible and practicable statewide research study.

We identified one newborn with SMA, consistent with incidence previously reported in the literature. False positive and inconclusive results due to out-of-range Cq values for *RPP30* have been observed in other studies [10] and state programs, even those that used higher cut-offs. This appears to be disproportionately true for babies with an NICU history [22]. We chose a conservative cut-off to detect specimens deviating from the Cq distribution of a healthy population and to investigate the causes of the out-of-range qPCR results. We detected one screen-positive result (Case #1) likely caused by an unrelated blood disorder. Both *SMN1* and *RPP30* Cq values were close to the cut-off, indicating lower qPCR efficiency and suggesting that a higher *SMN1* cut-off might be appropriate to increase the specificity of the assay used in this study.

Important differences exist between public health NBS and opt-in studies such as EC. The challenges experienced in opt-in studies frequently affect the timeline from the baby’s birth to obtaining actionable results required for initial clinical evaluation and intervention. In addition to the same delays experienced by state NBS programs, such as delays due to holidays, staffing, equipment issues, sample collection, and shipping, reporting of results in EC was also delayed because of (1) postnatal enrollment for the majority of participants, (2) the requirement that all state testing must be completed prior to EC testing, and (3) the fact that EC testing is not performed daily for all conditions.

The difference between obtaining actionable results for pre- and postnatally enrolled babies could be critical for conditions such as SMA, where babies benefit the most if treatment is initiated presymptomatically [16,23]. The mean age of symptom onset is 2.5 months for SMA type I but can range from 1 to 11 months [24]. In the ongoing NURTURE [NCT02386553] trial of nusinersen treatment of presymptomatic infants with SMA type I treatment was initiated by 6 weeks of age and in the SPR1NT [NCT03505099] trial of onasemnogene abeparvovec-xioi by 42 days of age. Although the majority of results for babies enrolled in EC, both pre- and postnatally, were reported within this timeframe, further optimization of the enrollment and screening pipeline is needed to ensure that optional post state NBS is completed and a path to presymptomatic treatment is offered to all newborns in a timely manner.

## 5. Conclusions

Emerging transformative treatments such as those for SMA are expected to drive expansion of NBS panels in the coming years. Pilot studies play a critical role by demonstrating the feasibility of NBS and supporting the transition from recommendation for screening to ultimate implementation. As a part of this pilot study, we tested the validity of the screening assay over the screening period, feasibility of the screening and reporting algorithms, designed systems of short- and long-term follow-up care in the state, and assessed outcomes from a voluntary screening program using virtual recruitment strategies. We demonstrated that the system worked in identifying a child with SMA and facilitating a path to early treatment. Finally, our work provided the opportunity for all parents to choose screening for SMA prior to full statewide implementation.

## Figures and Tables

**Table 1:** Assay Cut-offs.

Testing Phase	Result	*SMN1* (Cq)	*RPP30* (Cq)
Initial (single punch)	Screen-negative	<26	- ^1^
	Cut-off for retest	≥26	- ^1^
Retest (duplicate)	Screen-negative	<27	-
	Screen-positive (ABN)	≥27	<26
	Unsatisfactory specimen	≥27	≥26

^1^ Qualitative assessment of *RPP30* was used for initial testing. Specimens with no *RPP30* amplification were retested in duplicate.

**Table 2:** Demographics for babies screened for SMA by Early Check (*n* = 12,065).

Sex	% Total
Males	49.9
Females	48.2
Other/Unknown	1.9
**Birthweight**	
Normal > 2500	94.1
Low 1500–2500	5.3
Very low < 1500	0.6
Unknown	0
**Gestational Age**	
Full term > 37	72.5
Preterm 28–37	15.4
Extremely preterm < 28 weeks	0.2
Unknown	11.9

**Table 3:** Screen-positive testing and reporting timeline. Values are given in days after birth.

	Case #1	Case #2
Specimen collected	2	1
Specimen received by state	5	3
NBS results reported by state	6	7
Consent obtained by EC	17	−1
Specimen imported by EC	20	8
Results reported by EC	26	10
Clinical evaluation	30	13
Confirmatory testing	36 ^1^, 40 ^2^, 57 ^3^	19
Second clinical evaluation	53 ^4^	21 ^5^
AAV9 result	N/A	26
Treatment initiated	N/A	30 (nusinersen)
Carrier testing results for parents	63 ^6^	N/A

^1^ Confirmatory test (baby blood) failed; specimen did not meet quality standards for unknown reasons. ^2^ Confirmatory test (baby saliva) failed; specimen did not meet quality standards because of insufficient quantity. ^3^ Saliva kits sent to parents for carrier testing. ^4^ Hematology clinical evaluation. ^5^ Clinical evaluation and AAV9 testing. ^6^ One parent heterozygous carrier and one negative.

## Supplementary Materials

The following are available online at https://www.mdpi.com/2409-515X/7/1/20/s1, Figure S1: SMA assay validation population ranges; Table S1: Total precision, Table S2: Reference range; Table S3: Factors affecting time to testing and reporting for babies screened for SMA by EC.
