# Supplementary material for: A Voluntary Statewide Newborn Screening Pilot for Spinal Muscular Atrophy: Results from Early Check
Source: Int J Neonatal Screen. 2021 Mar 21;7(1):20. doi: 10.3390/ijns7010020 (PMC8006221; doi:10.3390/ijns7010020)
Supplement: Supplementary file 1 [file IJNS-07-00020-s001.pdf]

Supplementary Materials

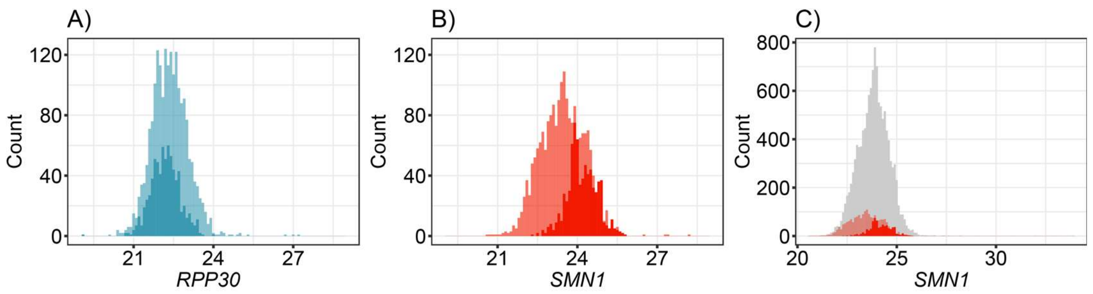

Figure S1. SMA assay validation population ranges. Overlays of Cq histograms from initial (n = 1982) and second (n = 736) population studies for *RPP30* (A) and *SMN1* (B) with distinct sample sets from routine NBS specimens obtained from the NCSLPH. The lighter bars are from the larger initial validation dataset, light blue in (A) and light red in (B) and opaque bars are from the smaller second validation dataset, dark blue in (A) and dark red in (B). (C) *SMN1* Cq as in (B) from initial and second validation overlayed with all *SMN1* screening results (gray, n = 12,065).

Table S1. Total Precision.

**Initial Validation**						
**Quality Control Type**	***SMN1* (Cq)**			***RPP30* (Cq)**		
	**Mean**	**SD**	**%CV**	**Mean**	**SD**	**%CV**
Normal	25.48	1.18	4.63	23.84	0.79	3.33
SMA patient (PT7)	No Cq	-	-	21.14	0.69	3.29
SMA Carrier (Car8)	24.64	0.75	3.05	22.37	0.8	3.58
**Second Validation**						
**Quality Control Type**	***SMN1* (Cq)**			***RPP30* (Cq)**		
	**Mean**	**SD**	**%CV**	**Mean**	**SD**	**%CV**
Normal	25.62	0.42	1.65	23.16	0.4	1.65
SMA patient (PT7)	No Cq	-	-	21.29	0.56	2.64
SMA Carrier (Car8)	24.83	0.53	2.15	22.23	0.46	2.07

Table S2. Reference Range.

		SMN1 (Cq)					*RPP30* (Cq)				
**Validation**	**n**	**Mean**	***SD***	***Min***	***Max***	***99.9%***	***Mean***	***SD***	***Min***	***Max***	***99.9%***
Initial	1982	23.44	0.84	20.64	28.16	27.31	22.36	0.68	20.15	27.25	26.73
Second	736	24.17	0.56	22.33	25.81	25.74	22.19	0.54	19.14	24.05	23.71

Table S3. Factors affecting time to testing and reporting for babies screened for SMA by EC. (n = 12,065)

**Age at sample collection**	**% total**
≤1 day post birth	0.8
1 day post birth	70.3
2 days post birth	25.2
>2 days post birth	3.7
**DBS received by the state laboratory**	
<3 days post birth	16.8
3 days post birth	38.4
4 days post birth	23.7
5 days post birth	12.7
>5 days post birth	8.4
**Results reported by the state laboratory**	
<4 days post birth	8.1
4 days post birth	21.1
5 days post birth	23.5
6 days post birth	25.5
7 days post birth	13.2
>7 days post birth	8.5
